# Editorial and Miscellaneous

**Published:** 1864-11

**Authors:** 


### EDITORIAL AND MISCELLANEOUS

*Rush Medical College.*—The twenty-second course of instruction in this Institution was commenced on the 5th of October. A very pleasant audience of ladies and gentlemen filled, to its utmost capacity, the lower lecture-room of the College building, on the evening of that day, to listen to the Introductory Address, by Prof. Miller. The numerous Alumni of the school, and its many staunch friends, will be glad to know that the current session promises to be more successful than any that has preceded it. The present attendance numbers between two and three hundred, and in intelligence and attainments, the class of students will compare favorably with any that we ever looked upon. The faculty are perfectly united and harmonious in their views as to the best plan for instructing medical students, and strengthened and encouraged by the approval their former course has received from the profession of the Northwest, they feel determined to put forth renewed efforts to sustain the reputation the school has acquired, and to give to the young men who annually avail themselves of its means of instruction, ample opportunities to gain a full knowledge of those fundamental principles that are essential in the education of a competent physician.

*Suspension of Medical Journals.*—The Rebellion and the financial derangements in the country, consequent thereupon, have operated most disastrously upon the Medical periodicals of the land. Not a few, indeed, a majority of the Medical journals published in 1860, have been compelled to suspend. One of the latest that has discontinued, is the *Medical Times* of New York city. This we regret, for the vacuum thus made in our exchange list, we fear, will remain unfilled. And we can but think that the profession of the Atlantic States, in allowing this journal to suspend, in consequence of inadequate pecuniary support, have done themselves a real injury. The difficulties of Medical journalism at all times, are not inconsiderable, and at times they accumulate so as to produce a stasis in the *circulation*. When the *Times* discontinues, and the *Surgical Reporter* must defer its issue for weeks, and the Boston *Journal* acknowledges embarrassments in its way, we can but feel thankful that, through the promptness of our subscribers, the *Medical Journal* of Chicago feels none of these annoyances, that have visited the Medical periodicals of the older Eastern cities. With two exceptions, we believe, the *Chicago Medical Journal* is the oldest Medical periodical in the United States, it having been published twenty-one years; and never was its list of paying subscribers so large as now.

---

*The New Surgeon-General of the United States Army.*—Dr. Joseph K. Barnes, who has been acting Surgeon-General for some months, has been appointed Surgeon General of the United States Army, vice Dr. Wm. A. Hammond.

---

*Officers of the American Dental Association.*—Prssident, Dr. J. H. McQuillen of Philadelphia; Vice Presidents, Drs. C. P. Fitch of New York, H. Benedict of Detroit; Corresponding Secretary, Dr. George W. Ellis of Philadelphia; Recording Secretary, Dr. J. Taft of Cincinnati; Treasurer, J. J. Weatherby of Boston.

*Surgeons in Captivity.*—The following is from the Richmond *Despatch* of recent date:

"The following officers, captured at Tupelo, Miss., some time since, were brought to this city yesterday, and committed to Libby Prison, Surgeon J. L. F. Garrison, 50th Ind., Asst. Surg. H. C. Cooper, 178th New York, Asst. Surg. John Little, 24th Missouri, and acting Asst. Surg. R. P. Kendle, U. S. A."

*A New Ambulance.*—Asst. Surg. Howard, U. S. A., has prepared a model of a new ambulance, combining many and important advantages and conveniences not possessed by any form of ambulance hitherto in use, yet having the merit of extreme simplicity, and not liable to get out of repair.

*A Brevet Worthily Bestowed.*—It is with pleasure that we announce that Surg. Richard S. Satterlee, Medical Purveyor, U. S. A., for the past ten years located in the city of New York, who ranked as Major, has, after receiving the brevet of Lieut. Colonel and Colonel, finally received that of Brigadier General, dating from September 2d. These honors, which are very worthily bestowed, have, as officially stated, been conferred "for diligent care and attention in procuring proper army supplies, as Medical Purveyor, and for economy and fidelity in the disbursement of large sums of money."

General Satterlee entered the army in 1822, and has been in the service ever since. Since the Rebellion broke out in 1861, his duties have been arduous and responsible.

The General comes from good stock, his father being among the first to volunteer in the Revolution; he was subsequently Captain in the first Regiment, Regular Infantry, of the United States (Hazan's Legion); was in many of the trying ordeals of those days, and died of a wound received at the battle of Brandywine.

Honors of this character are so sparingly conferred on our profession, that this evidence of appreciation will be received with gratitude by the Medical Corps of the Army, than whom there is no more faithful and able corps in the service of the Government.

There are others of the older army surgeons who, by their long, arduous and faithful labors, have well-earned increased rank, and we trust it will be bestowed.

---

At a late meeting of the Cincinnati Academy of Medicine, Dr. Blackman presented an extraordinary specimen of osteo-sarcoma, or enchondromatous tumor of the femur, measuring four feet in circumference, and weighing sixty-four pounds.

---

Jonathan Knight, M. D., late Professor of Surgery in the Medical Department of Yale College, died at New Haven, Conn., August 25th, in the seventy-fifth year of his age.

